# Distinct bacterial communities in tropical island aquifers

**DOI:** 10.1371/journal.pone.0232265

**Published:** 2020-04-30

**Authors:** Marek Kirs, Veljo Kisand, Craig E. Nelson, Tineill Dudoit, Philip S. Moravcik

**Affiliations:** 1 Water Resources Research Center, University of Hawaiʻi at Mānoa, Honolulu, HI, United States of America; 2 Institute of Technology, Tartu University, Tartu, Estonia; 3 Department of Oceanography and UH Sea Grant, Daniel K. Inouye Center for Microbial Oceanography: Research and Education, University of Hawaiʻi at Mānoa, Honolulu, HI, United States of America; University of Illinois at Urbana-Champaign, UNITED STATES

## Abstract

The groundwater biome is a poorly characterized habitat hypothesized to harbor uniquely diverse bacterial communities; the degree to which these communities differ from associated soils is a central question in environmental microbiology. We characterized the Bacterial community composition in 37 aquifer and 32 surface soil samples across the island of O‘ahu, Hawaiʻi. Several bacterial phyla (Acetothermia, Omnitrophica, Parcubacteria, Peregrinibacteria) relatively abundant in the aquifer samples were rare to absent in the soils. Immense bacterial diversity detected in the deep aquifers indicates that these environments are not as homogenous as expected, but provide various niches and energy sources for wide variety of bacteria. A small proportion of OTUs were widespread in all the basal (0.63%) and all the dike aquifer (0.31%) samples. However, these core bacteria comprised an average of 31.8% (ranging 16.2%-62.0%) and 15.4% (0.1%-31.5%) of all sequences isolated from the basal and dike aquifers respectively. Bacterial community composition correlated significantly with the sodium, sulfate, potassium, total dissolved solids, nitrate, conductivity, and pH in the basal aquifers, while phosphate and bicarbonate levels were also highly important when dike water samples were included in the analyses. This was consistent with high relative abundance of putative chemolithoautoroph taxa in the aquifer communities relative to soils. Targeted molecular and culture-based fecal indicator microbial analyses indicated good water quality of aquifers. The dominance of unique, deeply branching lineages in tropical aquifers emphasizes a large adaptive potential in O‘ahu’s aquifers; variability among groundwater samples suggests that aquifer habitats are surprisingly variable potentially harboring a variety of chemolithotrophic energy sources. Although parallel analyses of conventional and alternative indicators indicated good groundwater quality, this study calls for groundwater monitoring programs which would consider public as well as ecosystem health.

## Introduction

Globally, about 95% of liquid fresh water is terrestrial groundwater [1] and roughly half of the world’s population relies on groundwater as their main drinking water source [2]. Population growth and changes in climate are expected to negatively impact groundwater availability and quality [3–5]. Anthropogenic microbial and chemical contaminants in groundwater can pose serious direct health risks as well as negatively impact the integrity and functioning of groundwater ecosystems [3]. Compromised biological activity and ecosystem health will further compromise groundwater quality [6]. Although the importance of groundwater ecosystems is being recognized, the microbiological component of those systems is still largely neglected by the current monitoring programs and related policies.

Groundwater ecosystems, perhaps one of the least explored environments on earth, harbor microbial communities which have an important role in subsurface biogeochemical cycling and biodegradation [1, 7–12]. Several novel bacterial lineages in this ecosystem, including taxa with unusual chemoautotrophic pathways, appear to be unique to groundwater environments [13] and therefore may have potential utility in various areas of bio-technology. Microbial communities in these low-nutrient environments are extremely vulnerable to environmental change [9, 10], hence these communities can be used to predict and assess the effect of various stressors on groundwater ecosystem health and services [14–16]. This is particularly important for island communities which are isolated by the sea and have limited capacity, hence more vulnerable to impacts from human activity and climate change.

There is no meaningful alternative when water in island aquifers is compromised. In Hawai‘i it is estimated that roughly 99% of water used by households is taken from aquifers [17]. Currently water quality of Hawai‘i aquifers is evaluated for selected indicator bacteria (heterotrophic bacteria, total coliforms, *Escherichia coli*) by the City and County of Honolulu Board of Water Supply (BWS) as directed by the United States of America federal Groundwater Rule (GWR) 71 FR 65574 [18]. BWS conducts weekly sampling of selected wells to evaluate bacteriological as well as chemical source water quality. However, we don’t know what bacteria grow in O‘ahu’s aquifers, hence have no information on the current state od those deep aquifers. While the monitoring of aquifers for indicator bacteria is certainly valuable, the microbiological component of Hawaii's aquifers as well as on other islands in the Pacific has remained poorly characterized.

It is important to recognize that environment and public health are tightly coupled. As bacterial flora of aquifers in the Pacific islands has remained to date essentially unexplored, there is a need to characterize the current state (‘baseline’) of the islands' aquifer bacterial biome. This will enable us to explore (and eventually predict) the effect of environmental changes on aquifer ecosystem and develop appropriate management strategies, ergo improving current water quality monitoring programs and sustainable water resource management.

The overarching goal of this study was to bridge environmental and public health sciences to initiate and provide in-depth characterization of the bacterial microbiome of Hawaiian aquifers by focusing on the aquifers of O‘ahu island, the most populated island in the Hawaiian archipelago. The primary objective was to explore bacterial diversity and composition of O‘ahu’s aquifers and surface soil samples to: 1) examine linkage between the two environments, and 2) identify physicochemical parameters explaining the distribution of bacteria in the aquifers. A wider set of microbiological water quality indicators than that used for regulatory purposes was examined in parallel.

## Materials and methods

### Sample collection

**Fig 1:**
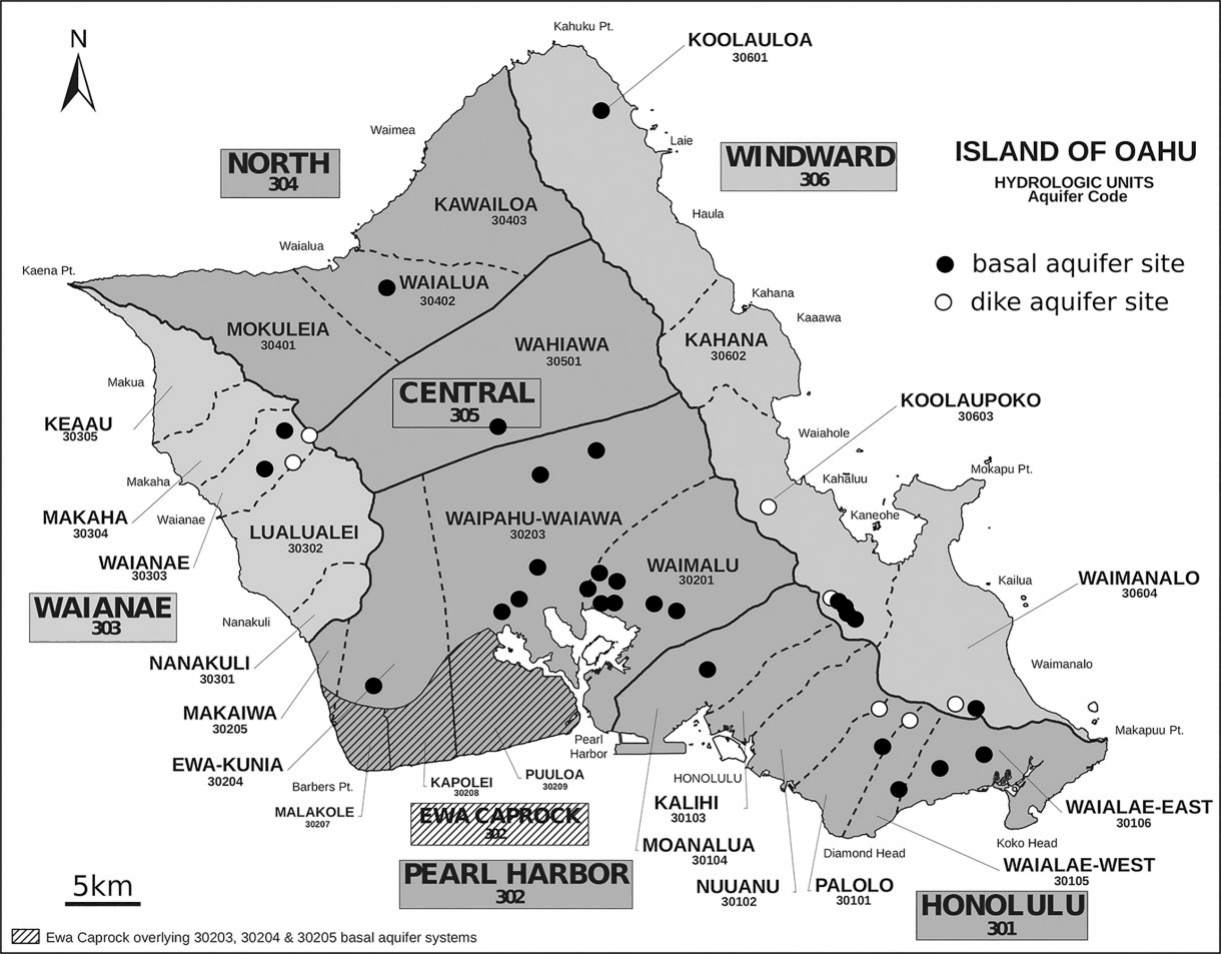
Groundwater collection sites. Groundwater from 30 basal aquifers and 7 dike aquifers was collected from the sites operated by the City and County of Honolulu Board of Water Supply. Base map by the Commission on Water Resource Management, Hawaiʻi.

Over a six-month period, from July 15^th^ to December 29^th^ 2017, nine liter of raw groundwater samples were collected from each of 37 wells used by the BWS to source water from the aquifers (Fig 1). The sites were selected by the BWS and each well was sampled once; all sites are fenced with tightly controlled access and not accessible to the public. The site access was granted and provided by the BWS who owns all the wells sampled. All major aquifers (Central, Honolulu, North, Pearl Harbor, Waianae, and Windward) (Fig 1) were sampled. A small subset of the sites (n = 7) were tunnels into the dike-impounded groundwater. Water collected from those sites is hereafter referred to as dike water. Pumps at the remaining 30 sites sourced water from basal aquifers through vertical wells (n = 28) or inclined shafts (n = 2). As purging of the wells is critical for obtaining samples truly representative of bacterial groundwater flora [19] pumps at each site were operated for a minimum of 20 minutes and sample outlets were sterilized and rinsed with groundwater for five minutes before sample collection. All samples were collected before chlorination. In addition to aquifer samples, at each site roughly 30–40 ml of soil was also extracted using sterile centrifuge tubes, and these were placed into sterile Whirl-Pak® bags. The samples were cooled and transported to the laboratory where they were processed within four hours of collection.

### Water chemistry

Water chemistry (concentrations of conductivity, alkalinity, total hardness, pH, turbidity, total dissolved solids, bicarbonate, calcium, chloride, magnesium, potassium, nitrate, phosphate, silica, sodium, and sulfate) data was analyzed and provided by the BWS according to standard operating procedures established for the state laboratory.

### Bacterial community composition analysis

In the laboratory, bacterial communities were concentrated from eight-liter sample volumes onto sterile hydrophilic polyethersulfone membrane filters (Supor®200, 0.2 μm pore size; Pall Corp., Ann Arbor, MI). Bacterial community DNA was recovered from the filters, and from 0.3 g of homogenized soil samples using DNeasy® PowerSoil® Kit (Qiagen; Germantown, MD) according to the manufacturer’s protocol, replacing vortexing with two minutes of bead beating at maximum speed on a Mini Beadbeater^™^ (Biospec Products Inc;. Bartlesville, OK). All DNA samples were eluted in 100 μl of elution buffer (10 mM Tric-HCl) during the final DNA recovery step. The sequencing library, covering the V3 and V4 regions of bacterial 16S RNA ribosomal gene was prepared according to the Illumina protocol [20] from the samples and sequenced on a MiSeq sequencer (Illumina, San Diego, CA) in a single run using V3 chemistry at the Advanced Studies in Genomics, Proteomics and Bioinformatics at the University of Hawaiʻi. Raw sequence reads and related metadata were deposited at the Sequence Read Archive (Bioproject# PRJNA494450, National Center for Biotechnology Information, U.S. National Library of Medicine). Demultiplexed sequences were filtered to remove low quality reads (Q<20) using Trimmomatic v.0.36 [21]. R1 sequences were clustered at 97% identity using VSEARCH pipeline [22], and aligned and compared to the reference database SSU Ref NR 119 (http://www.arb-silva.de/projects/ssu-ref-nr/) using the SINA aligner [23]. OTUs represented by ≤3 sequences were removed for alpha diversity analyses and OTUs observed in only one sample were removed from the beta diversity based analyses. In addition, all sequences of *Ralstonia*, known microbial contaminant of DNA extraction and PCR amplification kits [24], were removed from the analyses. Alpha rarefaction (S1 Fig), and beta diversity were evaluated using vegan (http://vegan.r-forge.r-project.org/) community analysis packages in the R statistical software environment (https://www.r-project.org/). Relative abundance of OTUs as represented by proportion of sequences of each OTU detected in each sample were log transformed for principal coordinate analyses (PCoA). The envfit function in vegan was used to identify significant parameters determining the distribution of OTUs in the samples. Co-occurrence networks were constructed for the groundwater samples at OTU as well as at phyla, class, and order rank as earlier described [25] utilizing R scripts available at the github (https://github.com/RichieJu520/Co-occurrence_Network_Analysis). Only the groups which were detected at least 40% of the samples were included in the co-occurrence analyses. From those, only the groups that exhibited significant (P < 0.01) strong (Spearman’s rank coefficient ≥0.6) correlation were exported as a GML network. The networks were visualized and edited in Gephi-0.9.2 [26].

### Fecal indicator organisms

Total coliform, *E*. *coli* and enterococci concentrations in groundwater were determined using Colilert-18® and Enterolert® in Quanti-Tray®/2000 (IDEXX Laboratories, Inc.; Westbrook, ME) respectively, according to the manufacturer’s protocol. Concentrations of *Clostridium perfringens*, a bacterium recommended to be used as a sewage tracer [27], were determined using membrane filtration-based methods which included incubation of filter membranes (GN-6, 0.45 μm pore size; Pall Corp., Ann Arbor, MI) on mCP media [28] in GasPak^™^ EZ Anaerobe Pouch System (BD Diagnostics; Franklin Lakes, NJ) at 42°C for 24 hours; the phosphatase test (20 seconds of exposure to ammonium hydroxide vapors) was used to confirm positive *C*. *perfringens* colonies as indicated by pink, red, magenta color reactions. Concentrations of F+ specific coliphages were determined for the groundwater samples using the single agar layer method as specified in USEPA Method 1602 [29] using 10 mL sample volumes and *E*. *coli* F_amp_ as a host. The F+ specific coliphage isolates were not tested for sensitivity of RNAase, hence concentrations determined are indicative of the F+ specific coliphage group in general. Indicator bacteria concentrations in soil samples were determined by vortexing 10 g of homogenized soil sample in 100 ml sterilized Milli-Q® (MilliporeSigma; Burlington, MA) water and analyzing samples as above, except coliphage concentrations were not determined.

### Molecular sewage markers

Groundwater samples (300 mL) were adjusted to a pH of 3.5 to ensure the adsorption of human polyomaviruses to the filters [30]. The samples were filtered through a mixed cellulose ester membrane filter (Pall Corporation, Ann Arbor, MI). The DNA was extracted from the filters as above. Soil samples were not tested for molecular markers. Concentrations of human-associated *Bacteroides* and human polyomavirus markers were determined using previously published primers and probes [30, 31] synthesized by Integrated DNA Technologies (Coralville, IO). Each 25 μl polymerase chain reaction (PCR) contained 5 μl of DNA sample, bovine serum albumin (0.2 mg mL^-1^ final concentration), forward and reverse primers (500 nM final concentration each), probe (80 nM final concentration) and SsoAdvanced^TM^ Universal Probes Supermix (diluted to 1X final concentration). Each sample was tested in duplicate on a CFX96 Touch^™^ Real-Time PCR Detection System (Bio-Rad Laboratories Inc., Hercules, CA). The cycling parameters in each PCR run were as follows: initial polymerase activation for two minutes at 95°C, followed by 40 denaturation cycles for ten seconds at 95°C and annealing-extension for 30 seconds at 60°C. The linearized plasmid, containing a target specific insert, was quantified using dsDNA HS Assay Kit on Qubit® 2.0 fluorometer (Life Technologies; Carlsbad, CA) and serially diluted. Triplicate reactions of each dilution were included in each quantitative PCR (qPCR) run. The samples containing PCR inhibitors were determined by challenging PCR reactions containing equal concentrations of salmon testes DNA (Sigma-Aldrich; St. Louis, MO) with 5 μl of an unknown sample [32]. Samples for which the threshold cycle was delayed by more than three PCR cycles when compared to reactions challenged by molecular grade water (corresponding roughly to a one log underestimate of initial concentration) were considered inhibited and subjected to a ten-fold dilution with molecular grade water.

## Results

### 1. Groundwater vs soil microbiome

**Fig 2:**
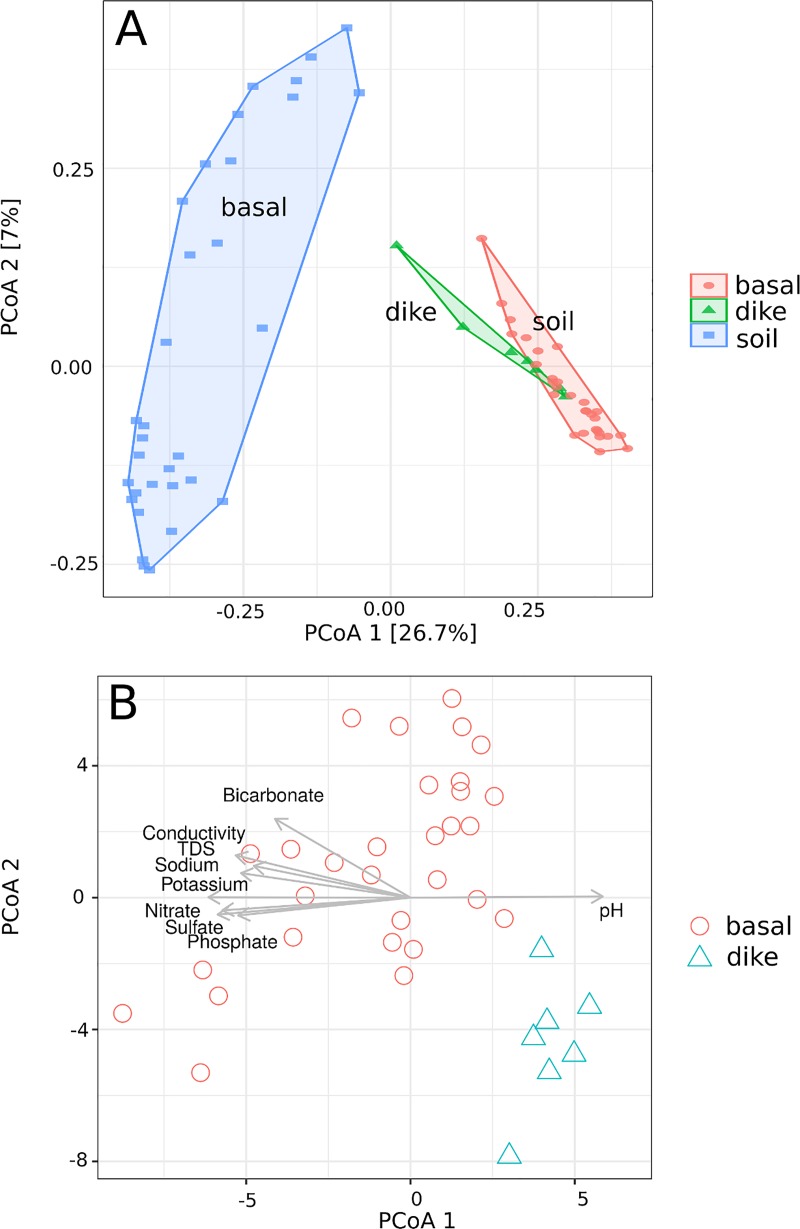
Principal coordinate analyses of bacterial communities in Oahu aquifers. Principal coordinate analyses of bacterial communities identified from the soil, basal aquifer and dike aquifer samples (A). Significant (P<0.001) environmental parameters, determined using envfit function in vegan, are shown as red vectors for basal and dike aquifer samples (B).

**Fig 3:**
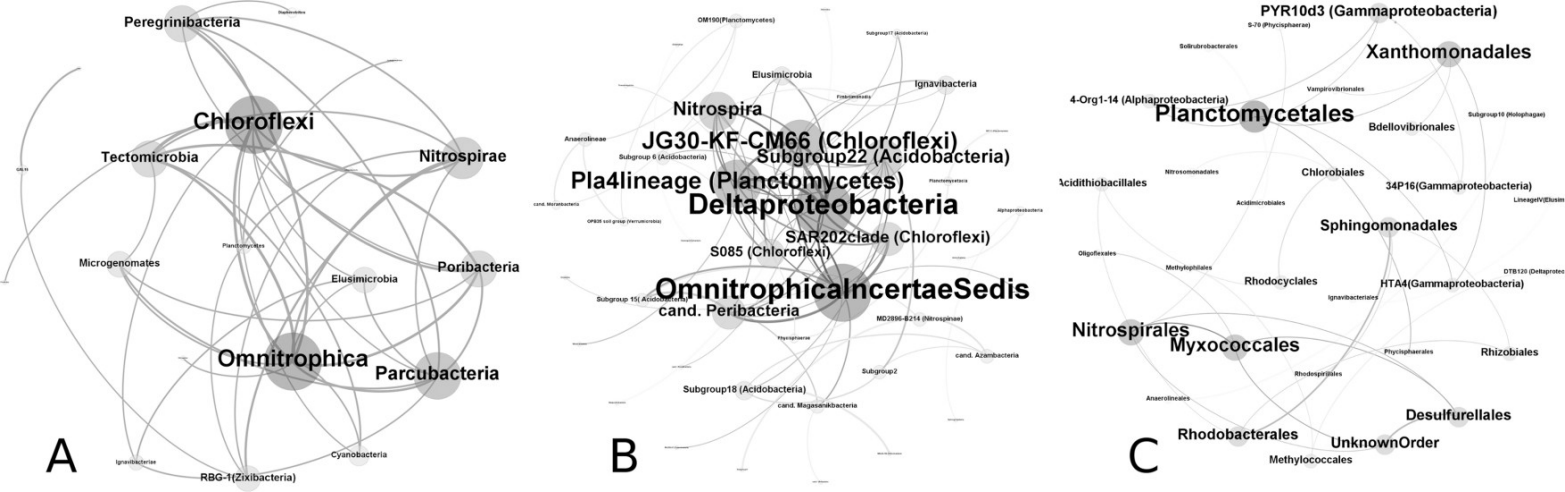
Co-occurrence network analyses of bacterial communities in Oahu aquifers. Co-occurrence networks of phyla (A), classes (B), and orders (C) detected in at least 40% of aquifer samples. Node size indicates relative proportions of co-occurring nodes (node degree). OTUs that could not be identified at given rank were excluded from the analyses.

Analyses of over 3.1 million bacterial sequences revealed that O‘ahu’s aquifers harbor structurally and functionally diverse bacterial communities (S2 Fig, S3 Fig, S4 Fig), the diversity of which is comparable, and in some samples exceeds, the microbial diversity found in adjacent surface soils (Table 1). While the diversity was found to be comparable in both sample types, the aquifer biome was very different from those found in soils (R^2^ = 0.41, P<0.001; Fig 2A). The aquifer samples were dominated by Betaproteobacteria (16.4%), Nitrospirae (12.4%) and Omnitrophica (12.3%) (S5 Fig), while Actinobacteria (22.9%), Alphaproteobacteria (18.3%), and Acidobacteria (14.6%) were predominant in the soil samples based on number of sequences detected in respective sample matrixes (S2 Fig, S3 Fig, S4 Fig and S5 Fig). Several bacterial phyla (Acetothermia, Omnitrophica, Parcubacteria, Peregrinibacteria) were associated with aquifer samples, but were rare to absent in soil samples (S4 Fig). From those, Omnitrophica and Parcubacteria, were more likely to co-occur with other bacterial phyla in the aquifer samples based on the co-occurrence analyses at phyla level (Fig 3). Roughly 3386 OTUs (12.5%) where shared by the soil and water samples indicating a limited link between the soils and aquifers. While some of the soil bacteria could certainly be transferred and adapt to aquifer environments, these energy depleted environments appear to select for very different bacterial groups.

**Table 1:** Average and range (in parenthesis) of sequence numbers, diversity, and evenness in the aquifer, and soil samples.

Sample type	Number of samples	Number of sequences	Richness	Diversity		Evenness
			OTUs per sample	Shannon’s (H)	Fisher α	J
Basal and dike	37	1751074	2071	5.37	468.9	0.71
			(185–4373)	(2.13–6.95)	(23.1–1257.2)	(0.41–0.85)
Basal	30	1413545	1956	5.33	424.8	0.71
			(185–4374)	(2.13–6.95)	(23.1–1257.2)	(0.41–0.85)
Dike	7	337529	2561	5.51	657.6	0.71
			(991–3274)	(3.51–6.31)	(196.5–706.0)	(0.48–0.80)
Soil	32	1407413	2397	5.98	571.6	0.78
			(190–3937)	(2.72–6.89)	(34.1–956.8)	(0.51–0.87)

### 2. Structural and functional bacterial diversity of O‘ahu groundwater

At OTU level, the dominant OTUs in the groundwater belonged to the Nitrospirae, Bacteroidetes, Chloroflexi, Proteobacteria phyla (S1 Table). Only 0.25% of all the OTUs in the groundwater aquifers could be identified to the genus level indicating a high degree of novelty.

To identify whether a core bacterial community exists, OTUs shared between the aquifer samples and between the aquifers were determined. Seventy OTUs (0.61%) were detected in all the samples collected from the basal aquifers and twenty eight OTUs (0.31%) were shared among all the dike water samples. Among those core OTUs, the top five most abundant OTUs based on the number of sequences detected, were identified as *Nitrospira*, *Sediminibacterium*, *Galionella*, an OTU from Omnitrophica phyla and an OTU from Chloroflexi (SAR202 clade) in basal aquifer samples (S2 Table). In dike water, two of the top five OTUs of the core community based on the number of sequences detected were identified as *Leptospirillum*, while other three were identified as *Cytophaga*, *Gallionella* and unidentified OTU from taxon 0319-6A21 currently classified within the order Nitrospirales. The full list of most abundant taxa in O‘ahu’s basal and dike aquifers is provided in the Supporting Information (S1 and S2 Tables). The number of OTUs shared among all samples dropped to 13 OTUs (0.09%) when both, the basal aquifer and dike water samples were pooled. While only a limited number of OTUs where shared, these OTUs comprised on average 31.8% (16.2%-62.0%) and 15.4% (ranging from 0.1%-31.5%) of all sequences isolated from basal and dike aquifer respectively. When basal and dike aquifers were pooled, the core sequences comprised on average 13.4% (ranging 0.03% - 21.8% among samples) of sequences in a given sample. Therefore, although a limited number of OTUs are associated with the aquifer bacterial core, these OTUs were abundant in most of the samples. An exception to this was a single water sample collected from Waimanalo Tunnel (0.1%) which was characterized by low diversity in general, although sufficient sequencing depth was clearly achieved (S1 Fig, sample W16).

Bacterial communities in the dike water were significantly different from the bacterial communities in the basal aquifers (R^2^ = 0.483, P<0.001) (Fig 2B). Dike water had lower cation and anion concentrations as well as higher pH than the basal aquifer water. Bacterial communities were different between the main six aquifers (R^2^ = 0.394, P = 0.025). Sodium, sulfate, potassium, total dissolved solids, nitrate, conductivity, and pH had most significant (P>0.01) relationship to microbial community structure among the parameters measured in the samples collected from the basal aquifers (S3 Table), while phosphate and bicarbonate levels were also highly important when dike water samples were included in the analyses (Fig 2B, S3 Table)

Although it is challenging to describe functional diversity based on partial 16S RNA gene amplicon sequencing data as some functions are restricted to a certain taxon or taxa while others vary among the groups, O‘ahu aquifers appears to contain functionally diverse bacterial communities. At least 12.43%, 0.92%, and 0.83% of sequences could be associated with putative nitrite oxidizers (*Nitrospira*, *Leptospirillum*, and others), ammonium oxidizers (uncultured Nitrosomonadaceae), and bacteria capable of fixing nitrogen respectively (*Azotobacter*, *Azospina* and others), while these percentages were 1.53, 1.47, and 4.23 for soil samples. Nitrite oxidizers appear to be an integral part of Hawai‘i aquifer communities, as four out of the five OTUs with the highest node degree (≥117, network of 11 484 nodes) belonged to Nitrospirales (0319-6A21 clade) (S6 Fig) based on the co-occurrence network analysis. Iron oxidizers (*Gallionella*, *Sideroxydans*, *Ferriphaselus*, *Ferritrophicum*, and closely related) were also important, with at least 4.33% of sequences being associated with bacteria capable of oxidizing iron. Sulfate oxidizers (*Sulfuricella*, *Sulfurifustis*, and closely related) and reducers (*Desulfovibrio*, *Desulfuromonas*, and others), as well as methano- and methylotrophs (*Methylobacterium*, *Methylomonas*, *Methylococcus*, and others) were also detected but at lower concentrations. Collectively at least 21.3% of sequences could be associated with putative chemolithoautotrophs in the aquifer and at least 10.47% in the soil samples. This is in agreement with the chemistry data discussed above, which indicated that nitrate, sodium, phosphate levels are important in explaining bacterial community variability in O‘ahu aquifers.

OTUs belonging to Flavobacteria, SAR202 clade, and an unidentified group of Bacteria had highest node degree (79 in a network of 996 nodes) in basal aquifers based on the co-occurrence network analysis (S6 Fig). While Flavobacteria and SAR202 clade could not qualify as keystone taxa based on high betweenness centrality (>5000) [33], the unidentified OTU appears to be important in Hawai‘i basal aquifers as indicated by high node degree and low betweenness centrality (4234). This OTU was never abundant, but present at low concentrations (<1% of sequences in given sample) and was never detected in the soil samples. While this OTU could not be identified at phylum level using the SINA aligner, it has 98% sequence similarity to an isolate (accession # JQ732832) from carbonate cave pools in New Mexico based on an online nucleotide Basic Local Alignment Search Tool (blastn) query of nucleotide collection (nr/nt) at the NCBI.

### 3. Microbial groundwater quality indicators

Fecal indicator bacteria indicated relatively good water quality of Oahu aquifers. Based on the cultivation data, six (four well water and two dike water samples) of the 37 aquifer samples (16.2%) were positive for total coliforms, nevertheless no *E*. *coli* or *C*. *perfringens* were detected in any of the water samples analyzed. No sewage-specific markers (human-associated *Bacteroides*, human polyomaviruses), or coliphages were detected in any of the aquifer samples. In contrast all soil samples were positive for total coliforms and enterococci. Geometric mean concentrations of both organisms were 1 930 MPN/g and 1 890 MPN/g, respectively, but frequently exceeded >2 419.6 MPN/g of soil (64% and 8% samples, respectively). *E*. *coli* was detected in 48.5% of the samples, and the concentrations varied from <1 to >2 419.6 MPN/g (geometric mean = 748 MPN/g). *C*. *perfringens* was detected in 39% of the soil samples (geometric mean = 50 CFU/g) and concentrations ranged from <1 to 620 CFU/g. These data further suggest that the link between surface soils and aquifers is limited.

## Discussion

High biodiversity is linked to functional stability and flexibility of ecosystems or habitats and indicates functional resilience [6, 34, 35]. This, partial 16S RNA gene amplicon sequencing-based study identified immense bacterial diversity in O‘ahu’s aquifers, which is comparable to, or even rivals the diversity found in surface soils. Average diversity estimates (Table 1) in Oahu aquifers, although not unusual for aquifer samples and heavily influenced by the analytical methods, appear to be elevated when compared to several studies [36–38] conducted in the continental aquifers. For example, in the studies referenced the average Shannon’s diversity index remained below 4, while in our samples the average index averaged roughly 5.3. The wide variety of taxonomical and functional groups detected likely indicates a large adaptive potential existing in O‘ahu’s aquifers.

Only a limited number of OTUs (<1%) were shared by all the groundwater samples. However these OTUs were abundant in the aquifer samples, ergo indicating importance of those core OTUs. In a recent study of deep crystalline rock aquifers in Finland [39], 0.4–4.1% of sequence reads belonged to OTUs shared by all groundwater samples, while in our study the proportion of core community OTUs ranged from 18.1% - 62.0% in basal (excluding dike water) aquifer samples.

O‘ahu aquifers harbor diverse bacterial communities. Low levels of biodiversity are typically associated with constant conditions, while greater biodiversity is typically associated with fluctuating environments [40]. However, in oligotrophic environments such as groundwater aquifers, variations of limited resources at scale of microbial cells can drive nutrient competition, leading to greater diversity [41–43]. The link to surface soils, which could also serve as potential source of diversity, appears to be limited as only 12.5% OTUs were shared between both environments, and several main phyla (Acetothermia, Omnitrophica, Parcubacteria, Peregrinibacteria) associated with groundwater samples were detected in only in few soil samples. Perhaps environmental conditions in O‘ahu aquifers aren’t as uniform and constant as one might envision, but provide various niches and energy sources for a wide variety of bacteria which have been accumulating and evolving over the millennia.

Inferring microbial functionality based on taxonomic composition is challenging as some functions are restricted to given taxa, while other functions are widespread across diverse groups of organisms [14]. Also taxonomic resolution obtained by analyzing the 16S RNA gene amplicon data is limited due to the short length of DNA sequence fragments. Nevertheless, these data do provide some indication of putative functional diversity present within the aquifer communities. The most dominant OTU in O‘ahu aquifers, 0319-6A21 belonging to order of Nitrospirales, first isolated from desert soils in Australia [44], but was recently detected at high abundance in microbial mats in California lava caves [45], perhaps indicating similarity between the available energy sources. Several other core taxa in O‘ahu’s groundwater such as *Gallionella*, *Nitrospirae* are frequently associated with aquifer environments [46, 47] and can harbor geochemically and geographically distinct lineages [48]. The SAR202 clade, was consistently detected in the aquifer samples, where it comprised up to 10.6% (average 3.5%) of all sequences. Although this group is typically associated with dark, deep ocean environments [49], a freshwater cluster has been described from Crate Lake (Oregon) [50]. The SAR202 clade is capable of organo- and lithotrophic metabolism, and its members are implicated as key players in the sulfur cycle in deep marine environments [49], a characteristic that could also hold true for the aquifer environments. While at least 12.5% of sequences analyzed from aquifer samples were attributed to chemolitoautotrophs, certainly various other energy sources are utilized by bacteria. In this regard, Peregrinibacteria, likely a group of endosymbionts [51], were almost exclusively detected in the aquifer samples, where it comprised about 1.3% of sequences analyzed (compared to 0.006% of soil samples).

Except for competition and predation, there is nothing to limit the growth of fecal indicator bacteria (sometimes also referred as pathogen indicator bacteria) in nutrient rich, moist, and warm edaphic environments in the tropics and subtropics. In this study fecal indicator bacteria were consistently recovered from soil samples. The proportion of *E*. *coli* and enterococci positive soil samples (100% and 48.5% respectively) was comparable to those reported earlier (95% and 54% respectively) [52]. Concentrations of *E*. *coli* and enterococci varied but were similar to those reported earlier for soils in Hawai‘i [53]. Geometric mean concentrations tended to be somewhat lower than those found in an earlier study [54] investigating fecal indictor bacteria concentrations in Hawaii’s soils. The geometric mean concentrations found in the present study are likely underestimates, as a large proportion of the samples exceeded the upper limit of detection (2 419.6 MPN/g). It should also be noted that samples analyzed for this study were collected from fenced locations, hence humans and larger-sized wildlife were unlikely source of fecal indicators.

Groundwater in O‘ahu aquifers appears to be of relatively good quality as no regulatory (enterococci), alternative fecal indicator bacteria (*C*. *perfringens*, coliphages) or sewage specific markers (human associated *Bacteroides*, human polyomaviruses) were detected. However 16.2% of samples were positive for total coliforms and a single sample (2.7%) for *E*. *coli*. These positive samples could be: 1) due to the infiltration of bacteria from surface soils trough geological strata and/or 2) due to compromised wells, and/or 3) due to biofilms which may harbor indicator bacteria [55]. Fecal indicator bacteria are certainly abundant and can grow in Hawaiian soils [52, 56], while regrowth of fecal indicator bacteria in some biofilms (potable water distribution system, irrigation system) have been suggested [57, 58]. However, if the wells are continuously operating or purged properly, detection of biofilm bacteria should be limited. Unfortunately we were not able to sample biofilm to investigate this issue further. More intensive sampling, especially when conducted during rain events and/or during various stages of purging could help to pinpoint the source.

Although we were able to sample all the main aquifers and the effort was made to extend the sample data set, we could only collect a single sample from each of the 37 wells. Therefore, it needs to be recognized that this study provides a current snapshot of Bacterial communities living in deep aquifers across the island. More extensive studies of the aquifers, which would also include analyses of wider range of chemical parameters, are highly desired.

This study was intended as an in-depth characterization of Hawaiian aquifers, and it did demonstrate that these aquifers harbor diverse bacterial communities. As environmental pressures on groundwater microbiomes are expected to change over time further studies can explore the effect of these changes and their impact on the integrity of Hawai‘i’s aquifer microbiome. The bacterial aquifer biome and human health are tightly coupled on many different levels, hence both—the environmental and human health need to be considered by monitoring programs focused on evaluating groundwater resources.

## Supporting information

S1 Fig Rarefaction curves for the soil (S*) and aquifer (W*) samples. (PDF)

S2 Fig Relative abundance (%) of main bacterial phyla and classes of Proteobacteria in the aquifer samples collected based on the number of sequences. Groups that comprised <1% of total sequences were clustered together as Others (<1%). CE–Central, HO–Honolulu, NO–North, PH–Pearl Harbor, WA–Waianae, WI–Windward aquifers. * indicates dike aquifers.
(PDF)

S3 Fig Main bacterial phyla and classes of Proteobacteria in the Oahu aquifers (water*) and soil (soil*) samples based on the number of sequences detected in each group. Aquifer samples includes both basal and dike (4, 5, 12, 14, 16, 19,20) aquifer samples.
(PDF)

S4 Fig Relative abundance of top ten phyla (A) and genera (B) in the aquifer (basal and dike) and soil samples.
(PDF)

S5 Fig Top ten classes (A), and genera (B) based on the sequence abundance in the Oahu aquifers (CN–central, HO–Honolulu, NO–North, PH–Pearl Harbor, WA–Waianae, and WI–Windward aquifers).
(PDF)

S6 Fig Co-occurrence networks of OTUs detected in at least 40% of groundwater (basal and dike aquifer combined) (A) and in at least 40% basal aquifer samples (B).
(PDF)

S1 Table Taxonomic affiliations of the most abundant Operational Taxonomic Units (OTUs). (PDF)

S2 Table Taxonomic affiliation of core OTUs that contributed >2% to core sequences. (PDF)

S3 Table Importance of various environmental parameters explaining bacterial community structure variations in samples collected from the aquifers and tunnels on Oahu island, Hawaiʻi. (PDF)
