# Supplementary figures and images for: Distinct bacterial communities in tropical island aquifers
Source: PLoS One. 2020 Apr 30;15(4):e0232265. doi: 10.1371/journal.pone.0232265 (PMC7192444; doi:10.1371/journal.pone.0232265)

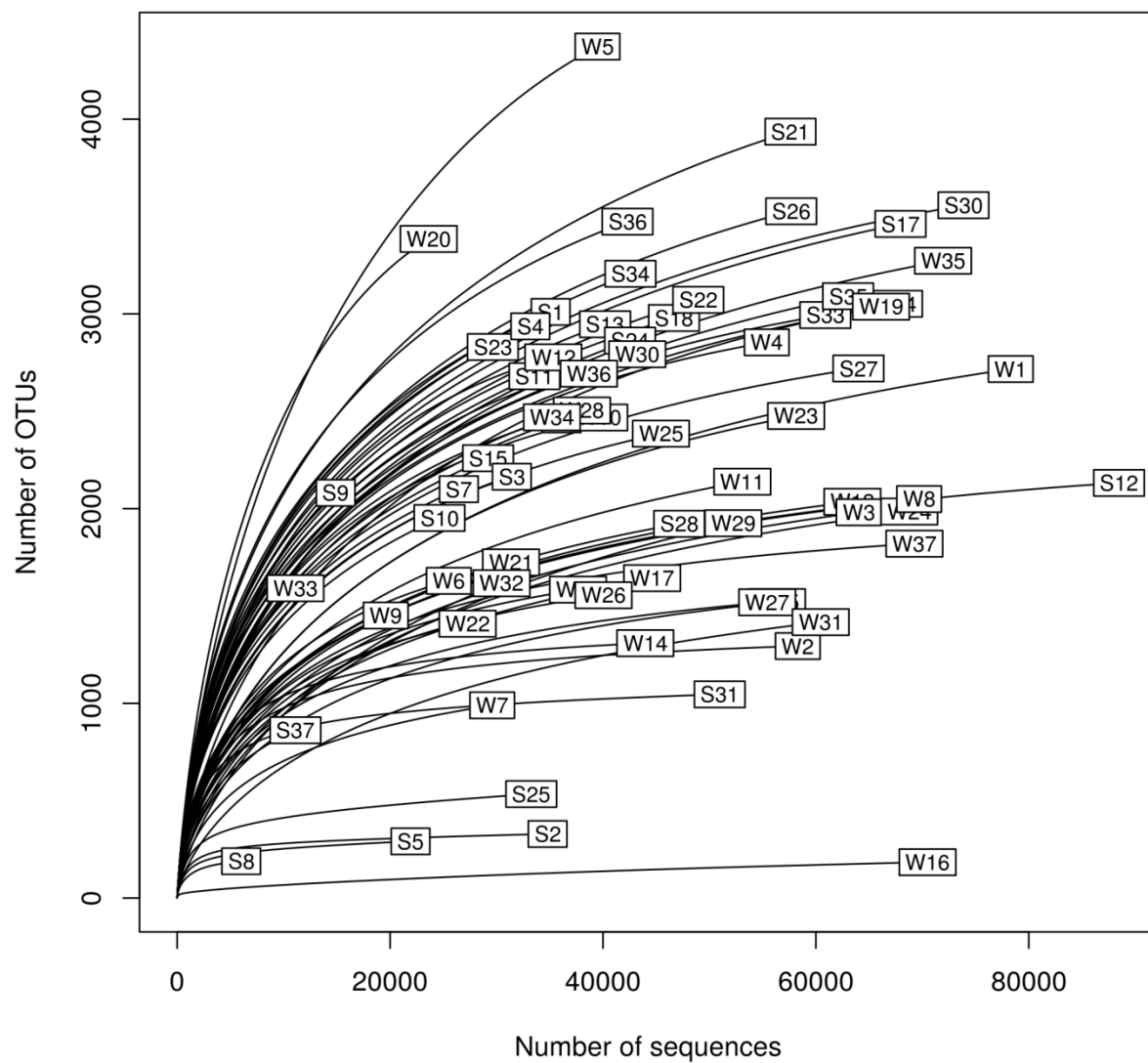

8

9 Figure S1. Rarefaction curves for the soil (S\*) and aquifer (W\*) samples.

Supplement: S1 Fig — (PDF) [file pone.0232265.s001.pdf]

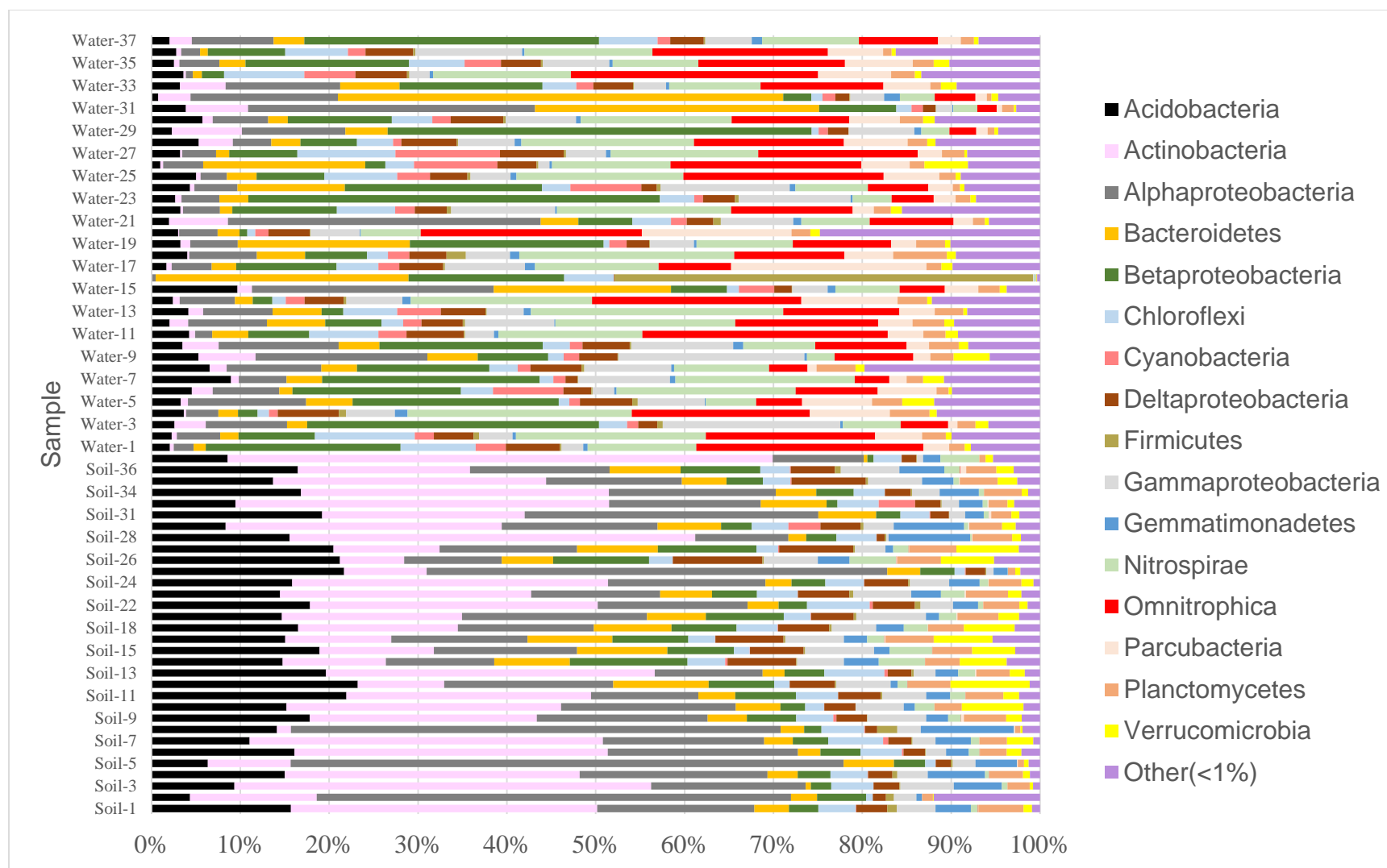

Supplement: S3 Fig — Aquifer samples includes both basal and dike (4, 5, 12, 14, 16, 19,20) aquifer samples. (PDF) [file pone.0232265.s003.pdf]

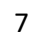

Supplement: S6 Fig — Co-occurrence networks of OTUs detected in at least 40% of groundwater (basal and dike aquifer combined) (A) and in at least 40% basal aquifer samples (B). (PDF) [file pone.0232265.s006.pdf]
