# Supplementary material for: Distinct bacterial communities in tropical island aquifers
Source: PLoS One. 2020 Apr 30;15(4):e0232265. doi: 10.1371/journal.pone.0232265 (PMC7192444; doi:10.1371/journal.pone.0232265)
Supplement: S2 Fig — Groups that comprised <1% of total sequences were clustered together as Others (<1%). CE–Central, HO–Honolulu, NO–North, PH–Pearl Harbor, WA–Waianae, WI–Windward aquifers. * indicates dike aquifers. (PDF) [file pone.0232265.s002.pdf]

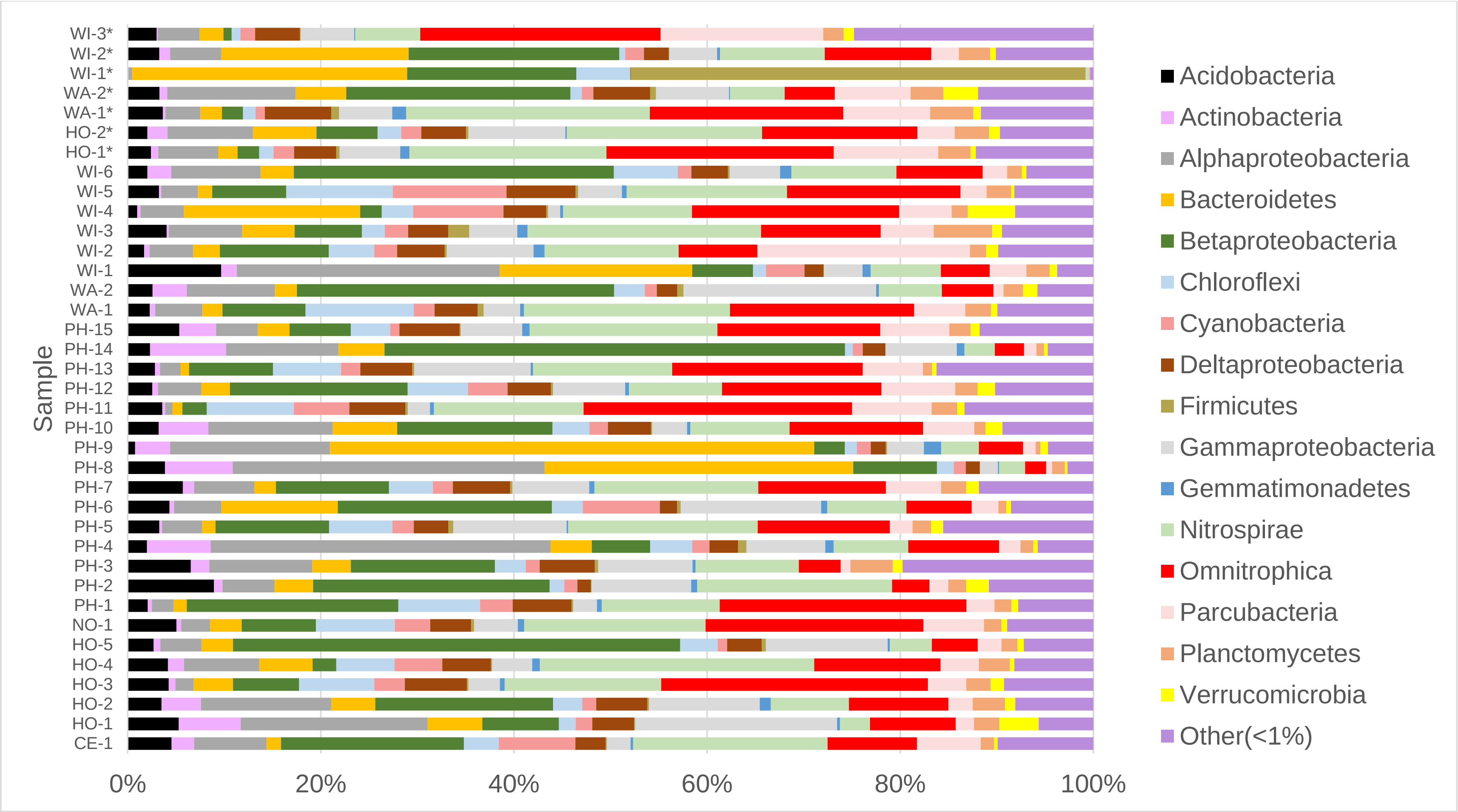

Figure S2. Relative abundance (%) of main bacterial phyla and classes of Proteobacteria in the aquifer samples collected based on the number of sequences. Groups that comprised <1% of total sequences were clustered together as Others (<1%). CE –Central, HO – Honolulu, NO – North, PH – Pearl Harbor, WA – Waianae, WI – Windward aquifers. * indicates dike aquifers.
