# Supplementary material for: Distinct bacterial communities in tropical island aquifers
Source: PLoS One. 2020 Apr 30;15(4):e0232265. doi: 10.1371/journal.pone.0232265 (PMC7192444; doi:10.1371/journal.pone.0232265)
Supplement: S4 Fig — Relative abundance of top ten phyla (A) and genera (B) in the aquifer (basal and dike) and soil samples. (PDF) [file pone.0232265.s004.pdf]

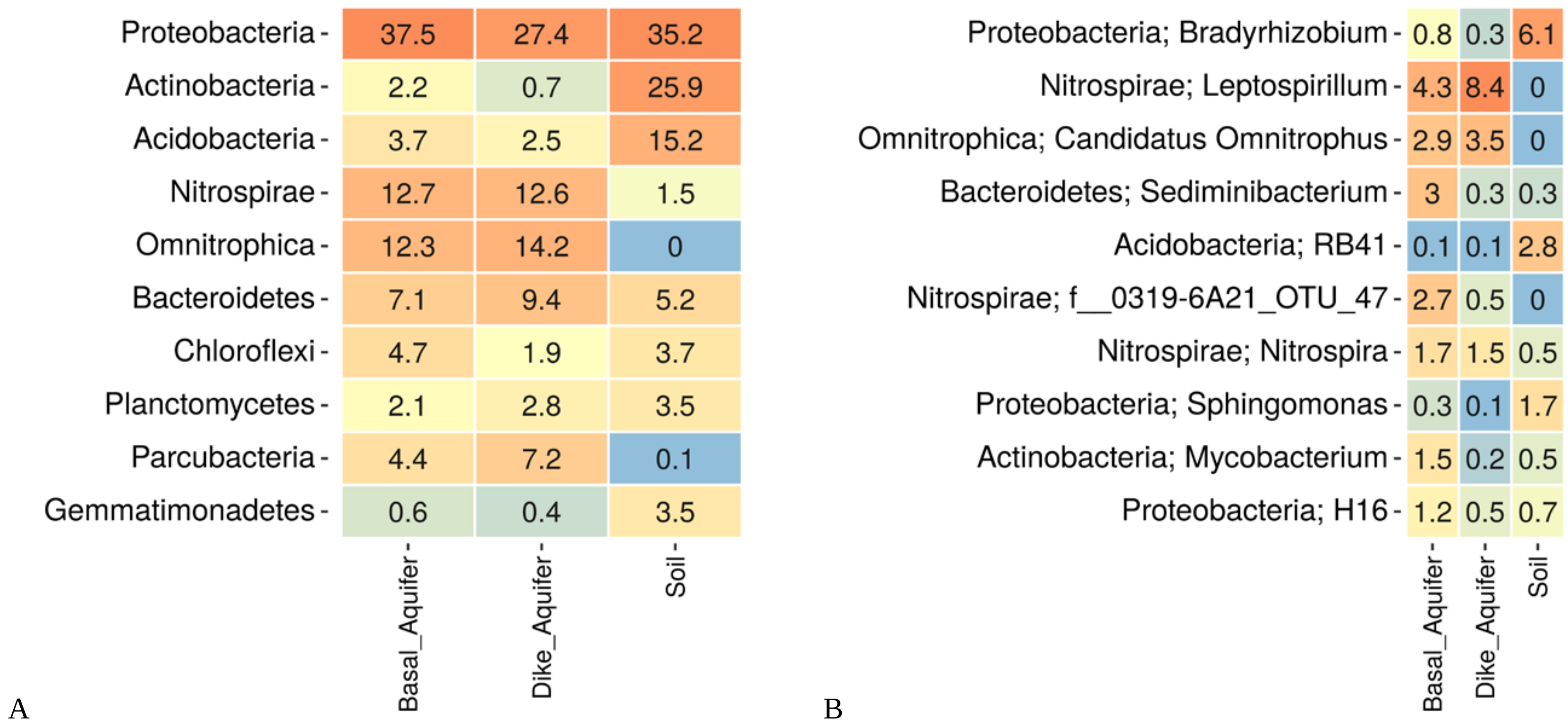

Figure S4 Relative abundance of top ten phyla (A) and genera (B) in the aquifer (basal and dike) and soil samples.
