# Supplementary material for: Distinct bacterial communities in tropical island aquifers
Source: PLoS One. 2020 Apr 30;15(4):e0232265. doi: 10.1371/journal.pone.0232265 (PMC7192444; doi:10.1371/journal.pone.0232265)
Supplement: S5 Fig — Top ten classes (A), and genera (B) based on the sequence abundance in the Oahu aquifers (CN–central, HO–Honolulu, NO–North, PH–Pearl Harbor, WA–Waianae, and WI–Windward aquifers). (PDF) [file pone.0232265.s005.pdf]

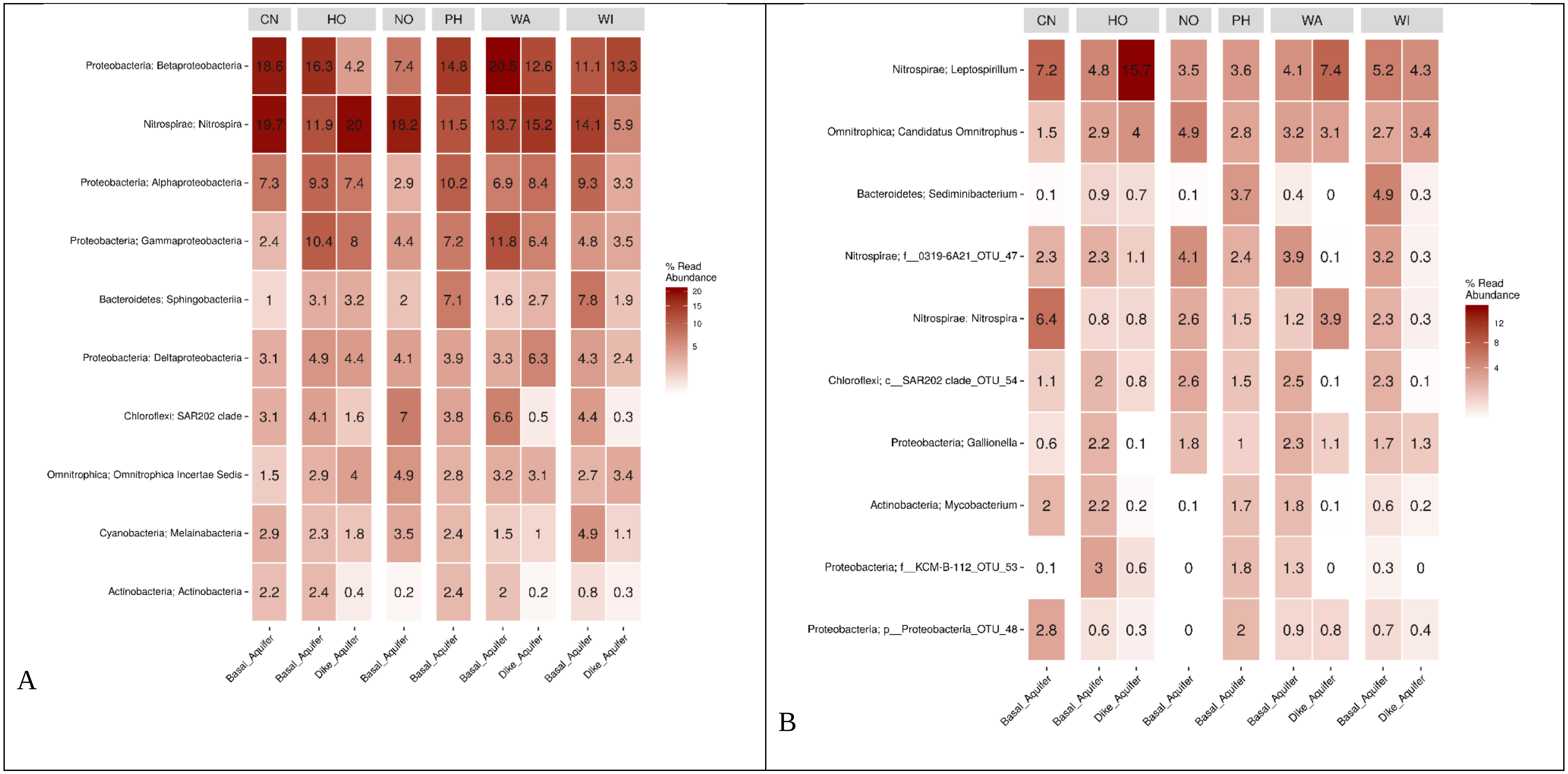

Figure S5 Top ten classes (A), and genera (B) based on the sequence abundance in the Oahu aquifers (CN – central, HO – Honolulu, NO – North, PH– Pearl Harbor, WA – Waianae, and WI – Windward aquifers).
