# Supplementary material for: Distinct bacterial communities in tropical island aquifers
Source: PLoS One. 2020 Apr 30;15(4):e0232265. doi: 10.1371/journal.pone.0232265 (PMC7192444; doi:10.1371/journal.pone.0232265)
Supplement: S1 Table — (PDF) [file pone.0232265.s007.pdf]

Table S1 Taxonomic affiliations of the most abundant operational taxonomic units (OTUs).

RANK	#OTU ID	Phylum	Class	Order	Family	Genus	# of sequences	%
Water Samples								
1	OTU_47	Nitrospirae	Nitrospira	Nitrospirales	0319-6A21		40 782	2.4
2	OTU_43	Nitrospirae	Nitrospira	Nitrospirales	Nitrospiraceae	*Leptospirillum*	40 228	2.3
3	OTU_2	Bacteroidetes	Sphingobacteriia	Sphingobacteriales	Chitinophagaceae	*Sediminibacterium*	34 110	2.0
4	OTU_54	Chloroflexi	SAR202 clade				28 092	1.6
5	OTU_124	Proteobacteria	Betaproteobacteria	Nitrosomonadales	Gallionellaceae	*Gallionella*	23 950	1.4
6	OTU_76	Omnitrophica					21 173	1.2
7	OTU_19	Proteobacteria	Betaproteobacteria	Nitrosomonadales	Gallionellaceae	uncultured	20 990	1.2
8	OTU_53	Proteobacteria	Gammaproteobacteria	Acidithiobacillales	KCM-B-112		20 799	1.2
9	OTU_17	Bacteroidetes	Bacteroidia	Bacteroidales	ML635J-40		19 366	1.1
10	OUT_48	Proteobacteria					17 818	1.0
Soil Samples								
1	OTU_6	Proteobacteria	Alphaproteobacteria	Rhizobiales	Bradyrhizobiaceae	*Bradyrhizobium*	56 062	4.1
3	OTU_506	Actinobacteria	Thermoleophilia	Solirubrobacterales	288-2		15 418	1.1
4	OTU_123	Proteobacteria	Alphaproteobacteria	Rhizobiales	Xanthobacteraceae		13 960	1.0

5	OTU_16	Proteobacteria	Alphaproteobacteria	Caulobacterales	Caulobacteraceae	uncultured	10 782	0.8
6	OTU_377	Actinobacteria	Rubrobacteria	Rubrobacterales	Rubrobacteriaceae	*Rubrobacter*	10 762	0.8
7	OTU_218	Proteobacteria	Alphaproteobacteria	Sphingomonadales	Sphingomonadaceae	*Sphingomonas*	9 921	0.7
8	OTU_460	Proteobacteria	Gammaproteobacteria	Xanthomonadales	Xanthomonadales		9 878	0.7
9	OTU_224	Proteobacteria	Alphaproteobacteria	Rhodospirillales	Rhodospirillaceae		6 853	0.5
10	OTU_419	Proteobacteria	Betaproteobacteria	Nitrosomonadales	Nitrosomonadaceae	uncultured	6 788	0.5
10	OUT_243	Acidobacteria	Blastocatellia	Blastocatellales	Blastocatellaceae (Subgroup 4)	RB41	6703	0.5
