# Supplementary material for: Distinct bacterial communities in tropical island aquifers
Source: PLoS One. 2020 Apr 30;15(4):e0232265. doi: 10.1371/journal.pone.0232265 (PMC7192444; doi:10.1371/journal.pone.0232265)
Supplement: S2 Table — (PDF) [file pone.0232265.s008.pdf]

Table S2. Taxonomic affiliation of core OTUs that contributed ≥2% to core sequences.

	OTU taxonomic affiliation					Percent of core sequences
OTU Rank	Phylum	Class	Order	Family	Genus	%
BASAL AQUIFER						
1	Nitrospirae	Nitrospira	Nitrospirales	0319-6A21		9.2
2	Bacteroidetes	Sphingobacteriia	Sphingobacteriales	Chitinophagaceae	*Sediminibacterium*	8.0
3	Chloroflexi	SAR202 clade				6.5
4	Proteobacteria	Betaproteobacteria	Nitrosomonadales	Gallionellaceae	*Gallionella*	4.8
5	Omnitrophica					4.2
6	Proteobacteria	Betaproteobacteria	Nitrosomonadales	Gallionellaceae	*Sideroxydans*	3.9
7	Chloroflexi	SAR202 clade				3.9
8	Proteobacteria					3.8
9	Cyanobacteria	Melainabacteria	Caenarcaniphilales			3.5
10	Proteobacteria	Alphaproteobacteria	Rhodospirillales	Rhodospirillales	*Reyranella*	2.7
11	Omnitrophica					2.4
12	Nitrospirae	Nitrospira	Nitrospirales	Nitrospiraceae	*Leptospirillum*	2.3
13	Proteobacteria	Alphaproteobacteria	Rhizobiales	Bradyrhizobiaceae	*Bradyrhizobium*	2.1

14	Proteobacteria	Betaproteobacteria	Burkholderiales	Comamonadaceae	*Aquabacterium*	2.1
15	Chloroflexi	SAR202 clade				2.0
DIKE AQUIFER						
1	Nitrospirae	Nitrospira	Nitrospirales	Nitrospiraceae	*Leptospirillum*	38.6
2	Bacteroidetes	Cytophagia	Cytophagales	Cytophagaceae	uncultured	23.1
3	Nitrospirae	Nitrospira	Nitrospirales	Nitrospiraceae	*Leptospirillum*	5.6
4	Proteobacteria	Betaproteobacteria	Nitrosomonadales	Gallionellaceae	uncultured	4.5
5	Nitrospirae	Nitrospira	Nitrospirales	0319-6A21		2.9
6	Proteobacteria	Alphaproteobacteria	4-Org1-14			2.9
7	Proteobacteria	Alphaproteobacteria	Rhodospirillales	Rhodospirillales	*Reyranella*	2.4
8	Proteobacteria					2.3
9	Cyanobacteria	Melainabacteria	Caenarcaniphilales			2.3
10	Proteobacteria	Betaproteobacteria	Nitrosomonadales	Gallionellaceae	*Sideroxydans*	2.2
