# Supplementary material for: Distinct bacterial communities in tropical island aquifers
Source: PLoS One. 2020 Apr 30;15(4):e0232265. doi: 10.1371/journal.pone.0232265 (PMC7192444; doi:10.1371/journal.pone.0232265)
Supplement: S3 Table — (PDF) [file pone.0232265.s009.pdf]

Table S3. Importance of various environmental parameters explaining bacterial community structure variations in samples collected from the aquifers and tunnels on Oahu island, Hawai‘i.

	Basal aquifer samples			All water samples		
	R^2	P	Significance[1]	R^2	P	Significance
Conductivity	0.416	0.005	**	0.370	0.006	**
Alkalinity	0.243	0.063	.	0.183	0.048	*.
Hardness	0.300	0.032	*	0.134	0.129	
pH	0.415	0.003	**	0.535	0.001	**
Turbidity	0.088	0.389		0.137	0.142	
Total Dissolved Solids	0.501	0.003	**	0.468	0.001	***
Bicarbonate	0.230	0.066	.	0.355	0.004	**
Calcium	0.209	0.101	.	0.126	0.147	
Chloride	0.316	0.019	*	0.217	0.040	*
Magnesium	0.295	0.035	*	0.231	0.029	*
Potassium	0.524	0.002	**	0.425	0.001	***
Nitrate	0.488	0.005	**	0.543	0.001	***
Phosphate	0.368	0.012	*	0.434	0.001	***
Silica	0.035	0.694		0.275	0.012	*
Sodium	0.679	0.001	***	0.591	0.001	***
Sulfate	0.575	0.001	***	0.515	0.001	***

[1]Significance codes: 0 ‘***’ 0.001 ‘**’ 0.01 ‘*’ 0.05 ‘.’ 0.1 ‘ ’ 1
